# Tuning Properties of MT and MSTd and Divisive Interactions for Eye-Movement Compensation

**DOI:** 10.1371/journal.pone.0142964

**Published:** 2015-11-17

**Authors:** Bo Cao, Ennio Mingolla, Arash Yazdanbakhsh

**Affiliations:** 1 Department of Psychiatry and Behavioral Sciences, Medical School, The University of Texas Health Science Center at Houston, Houston, United States of America; 2 Department of Communication Sciences and Disorders, Northeastern University, Boston, United States of America; 3 Center for Computational Neuroscience and Neural Technology, Boston University, Boston, United States of America; 4 Department of Psychological & Brain Sciences, Boston University, Boston, United States of America; Monash University, AUSTRALIA

## Abstract

The primate brain intelligently processes visual information from the world as the eyes move constantly. The brain must take into account visual motion induced by eye movements, so that visual information about the outside world can be recovered. Certain neurons in the dorsal part of monkey medial superior temporal area (MSTd) play an important role in integrating information about eye movements and visual motion. When a monkey tracks a moving target with its eyes, these neurons respond to visual motion as well as to smooth pursuit eye movements. Furthermore, the responses of some MSTd neurons to the motion of objects in the world are very similar during pursuit and during fixation, even though the visual information on the retina is altered by the pursuit eye movement. We call these neurons compensatory pursuit neurons. In this study we develop a computational model of MSTd compensatory pursuit neurons based on physiological data from single unit studies. Our model MSTd neurons can simulate the velocity tuning of monkey MSTd neurons. The model MSTd neurons also show the pursuit compensation property. We find that pursuit compensation can be achieved by divisive interaction between signals coding eye movements and signals coding visual motion. The model generates two implications that can be tested in future experiments: (1) compensatory pursuit neurons in MSTd should have the same direction preference for pursuit and retinal visual motion; (2) there should be non-compensatory pursuit neurons that show opposite preferred directions of pursuit and retinal visual motion.

## Introduction

**Fig 1:**
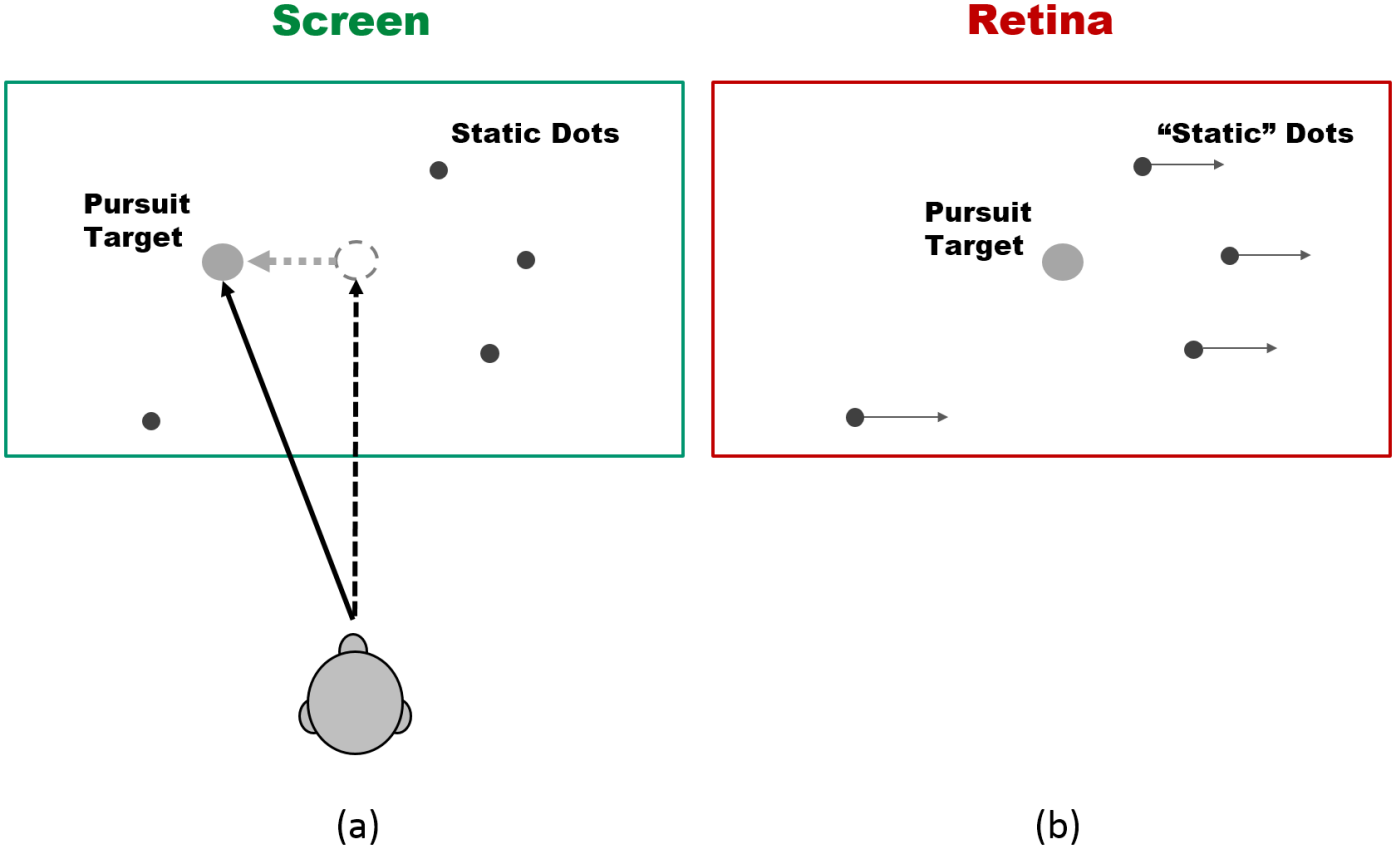
Screen and retinal images during the pursuit eye movement. The subject performs pursuit eye movements upon a target moving horizontally to the left on a screen with static background dots (a). On the retinal image during the pursuit, the target is relatively stable on the fovea, while the “static” dots are induced to move toward the right (b). Note that the normal inversion of the visual field on the retina is ignored in this diagram for the sake of clarifying the trade-off of retinal vs. screen motion during pursuit eye movement.

When we pursue a moving target with our eyes on a static background, the target is relatively static in the foveal region of our visual field. We can thereby further process detailed information about the moving target and proceed with actions, such as aiming or reaching for it with hands or tools. However, during a smooth pursuit eye movement, a static background will move in a direction opposite to the target in our visual field and thus will generate in and near the foveal region a similar retinal image as if the background were actually moving (Fig 1). So the local retinal information is difficult for our brain to distinguish in the following two scenarios: 1) when we perfectly pursue a target moving in one direction at certain speed on a static background at a fixed depth and 2) when we see a static target against a background surface that is moving in an opposite direction at the same speed. The brain uses both motor information about the eye movement and visual motion information from the retina to generate accurate perception and response to these two scenarios.

### Pursuit and visual motion responses of MSTd neurons

Monkey single-unit recording studies have found motion-sensitive neurons in the medial temporal area (MT) and medial superior temporal area (MST). These neurons usually show elevated activities for visual motion toward their preferred directions, while showing decreased activity for visual motion in the direction opposite to their preferred directions [1]. When probed with motion along the axis of their preferred direction, these neurons’ responses are also tuned to the speed of the visual motion. Thus, for each neuron sensitive to visual motion, its response is a function of the motion’s velocity, where positive velocity represents the neuron’s preferred motion direction and negative velocity represents the opposite of its preferred direction. Some MT neurons will respond to the retinal motion (referred to as “motion on retina”; the velocity of the motion on the retina will be referred to as “retinal velocity”), no matter if the retinal motion is caused by the motion of a physically moving object or induced by eye movements. Their responses to these two types of motions are not different [2,3] (but see [4] for MT neurons whose responses can be modulated by eye movements).

**Fig 2:**
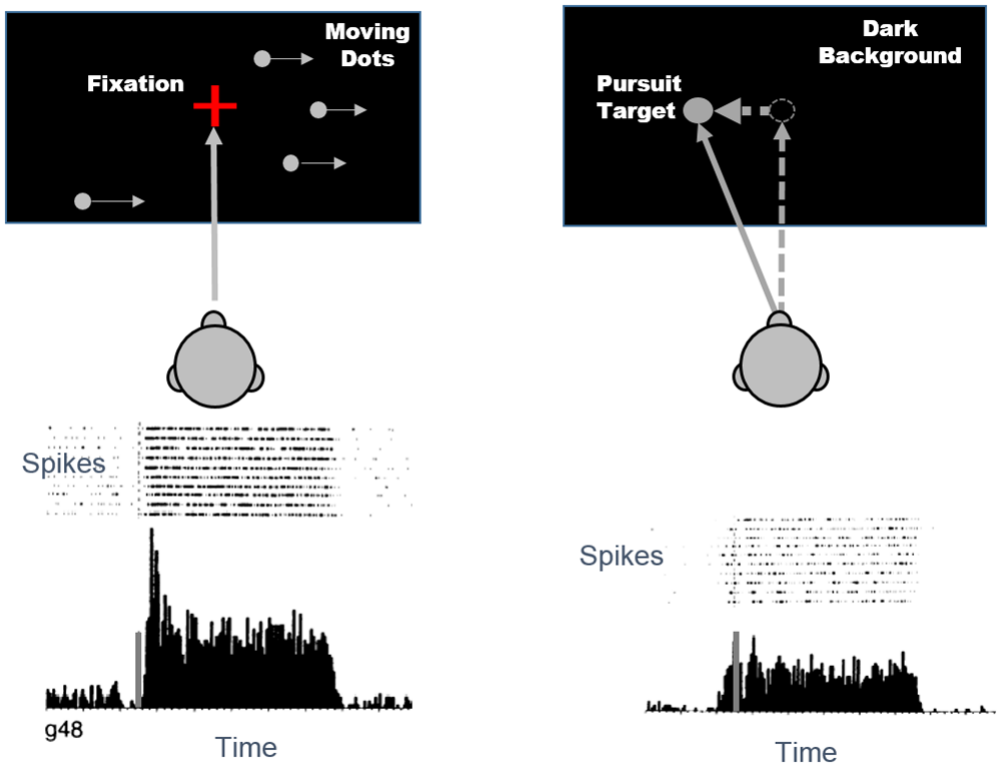
An MSTd cell that responds to both a large field dots moving to the right and to pursuit of a target moving to the left on a dark background. The height of the gray vertical bar indicates 250 spikes per second. Adapted from Komatsu and Wurtz (1988).

Other motion sensitive neurons, such as some neurons in the dorsal part of monkey medial superior temporal area (MSTd) respond to eye movements when the monkey pursues a target moving on a dark and homogeneous background, as well as when the monkey sees visual motion patterns, such as a large field of moving dots, while fixating (Fig 2) [5]. Some MSTd neurons can detect real motion (referred to as “motion on screen”, since the motion takes place on the computer screen; the velocity of the motion on screen will be referred to as “on-screen velocity”). These neurons adjust their responses for the component of motion on retina induced by eye movements [6].

### Pursuit compensation to represent the on-screen velocity in MSTd neurons

**Fig 3:**
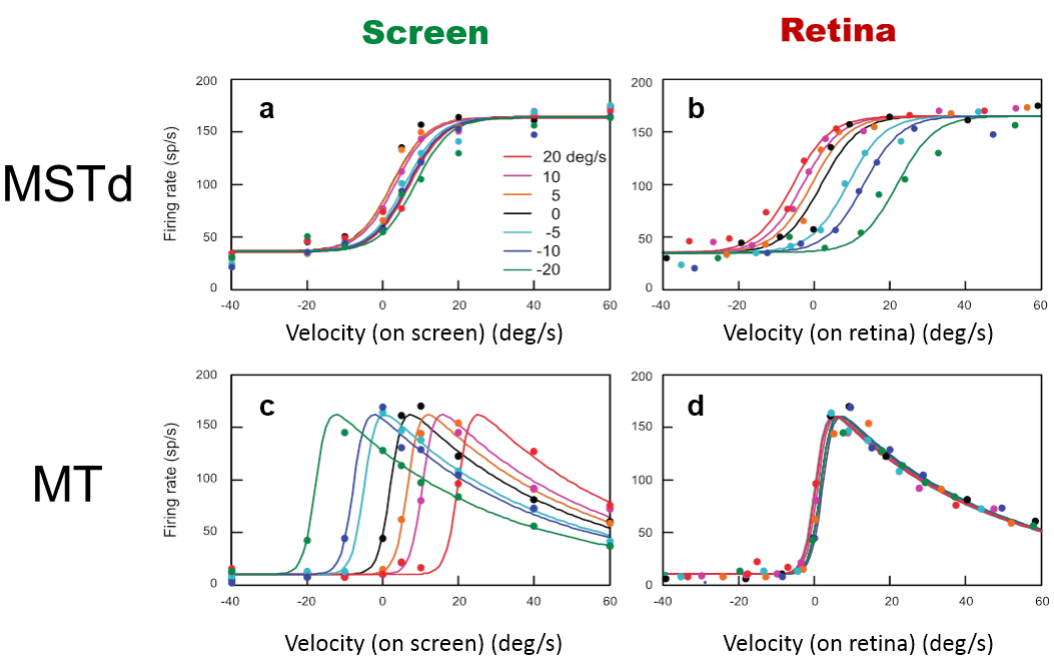
Pursuit compensation of MSTd neurons. MT neurons are tuned to retinal velocity. Thus, for different eye movement velocities, the velocity tuning curves of MT neurons shift away from the velocity tuning curve for the fixation condition in screen coordinates (c and d). Unlike MT neurons, MSTd neurons can compensate for the visual motion induced by pursuit to represent the real velocity on screen rather than the retinal velocity. The compensation is indicated by the overlapping velocity tuning curves in the screen reference frame for different eye movement velocities (a), as well as the shifting of the same curves in the retinal frame (b). The shifting distance of each curve depends on the pursuit speed. For a perfect compensation, the shift distance should be equal to the speed of the pursuit but in the opposite direction. Different colors represent different pursuit velocities as shown in the legends in (a). Adapted from Inaba et al. (2011).

The velocity tuning of MT and MSTd neurons support the idea that, unlike MT neurons that are tuned to retinal velocity, some MSTd neurons are tuned to the on-screen velocity of moving stimuli, even when there is a pursuit eye movement [6]. As shown in Fig 3c, for different pursuit eye movement velocity, the on-screen velocity tuning curves of MT neurons shift away from the velocity tuning curve for the fixation condition. However, the retinal velocity tuning curves overlap with each other regardless of the pursuit velocity (Fig 3d). This means that typical MT neurons are tuned to retinal velocity rather than on-screen velocity. Unlike MT neurons, some MSTd neurons can compensate for the visual motion induced by pursuit to represent the on-screen velocity rather than the retinal velocity, as shown in the overlapping curves of different pursuit velocities in Fig 3a. For example, for the same motion toward the neuron’s preferred direction on screen, pursuit in the direction opposite to the neuron’s preferred direction creates a larger speed of retinal motion than that during fixation. In retinal coordinates this larger retinal speed during pursuit generates a right shift of the velocity tuning (positive for the preferred visual motion direction) during pursuit relative to the velocity tuning during fixation (Fig 3b green/blue lines). However, for motion on screen, the velocity tuning of the MSTd neuron remains the same (green/blue lines in Fig 3a), compared to the response during fixation (black lines). The invariant velocity tuning for different pursuit velocity is a compensation for motion induced by pursuit (pursuit compensation). However, the computational mechanism underlying pursuit compensation is still an open question. We suggest one possible mechanism in the following paragraphs.

In this study, we develop a computational model of pursuit compensatory neurons in MSTd with simulated MT inputs. (1) The velocity tuning of our model MSTd neurons is similar to the velocity tuning of monkey MSTd neurons. (2) The model MSTd neurons also show the pursuit compensation property. We find that pursuit compensation can be achieved by divisive interaction between the eye-movement and visual motion signals combined with shunting computations. (3) We discuss the implications suggested by the model: (a) the compensatory pursuit neurons in MSTd should have the same direction preference of pursuit and visual motion and more generally have similar velocity tuning of pursuit and retinal visual motion. The model suggests that if a neuron in MSTd shows similar velocity tuning curves of pursuit and retinal visual motion, this neuron is a compensatory pursuit neuron; (b) there should be “non-compensatory pursuit neurons”. These neurons should show a modulation of responses during pursuit opposite to the modulation effect of pursuit in compensatory pursuit neurons. The model suggests that the neurons that show opposite preferred directions of pursuit and visual motion are in fact the non-compensatory pursuit neurons. (4) We also suggest that, on a single cell level, pursuit compensation responses to different visual motion and pursuit directions can be explained by a compensation mechanism that operates on the vector component of visual motion along the neuron’s preferred pursuit direction.

## Methods

Our model can account for two main properties of pursuit compensatory neurons in MSTd: a) the sigmoid shape of the velocity tuning curve, and b) the pursuit compensation response to visual motion. The model also provides implications that can be tested in physiological experiments (see discussion). In the following section we will demonstrate the computations of a sample model MSTd neuron. The output of this neuron shows both velocity tuning characteristic of MSTd neurons in vivo and the pursuit compensatory property.

### Summary of the Model

**Fig 4:**
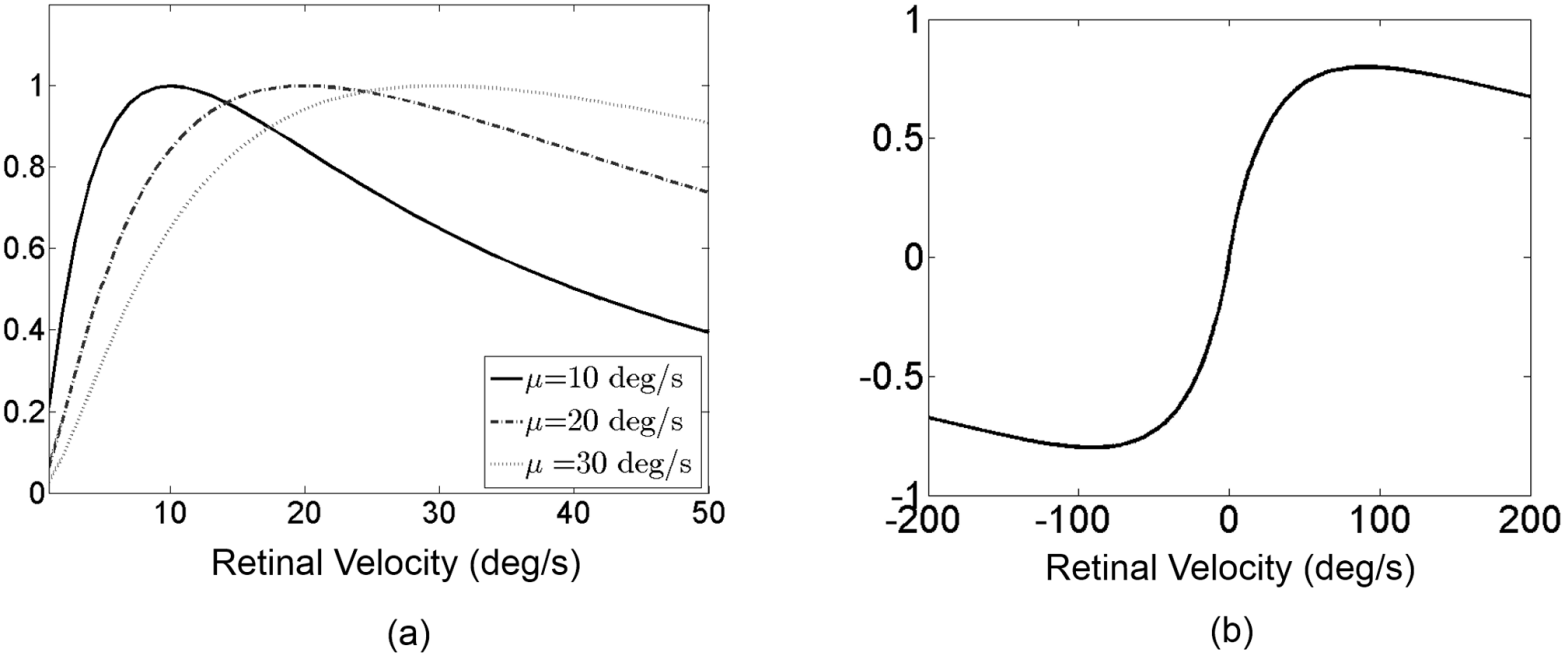
Model summary. The MSTd computation contains five stages: 1) calculate the summation of MT responses and calculate the exponential form of the MT summation, 2) define pursuit input as a function of pursuit velocity with the mirrored visual tuning function (see text), 3) calculate the result of divisive interaction between the MT and pursuit responses, 4) use the result as the input to a simple shunting computation and 5) solve the shunting equation in its equilibrium state.

The model is summarized in Fig 4. For each velocity *v*, the responses of the model MT neurons with different velocity preferences, *μ*, are summed with their corresponding weights, *w*_*μ*_ (Eq 1).

$$R_{MT}\left(v\right)=\Sigma_{{}_{\mu }}w_{\mu }R\left(\mu ,v\right)$$ (1)

where R is defined by following function to fit the speed tuning of single MT neuron with a speed preference of *μ* according to [7]:

$$R\left(\mu ,v\right)=\;e^{-\frac{{\text{log}}^{2}\left[q\left(\mu ,v\right)\right]}{2\sigma^{2}}}$$ (2)

$$q\left(\mu ,v\right)=\;\frac{v\;+\;s_{0}}{\mu \;+\;s_{0}}$$ (3)

**Fig 5:**
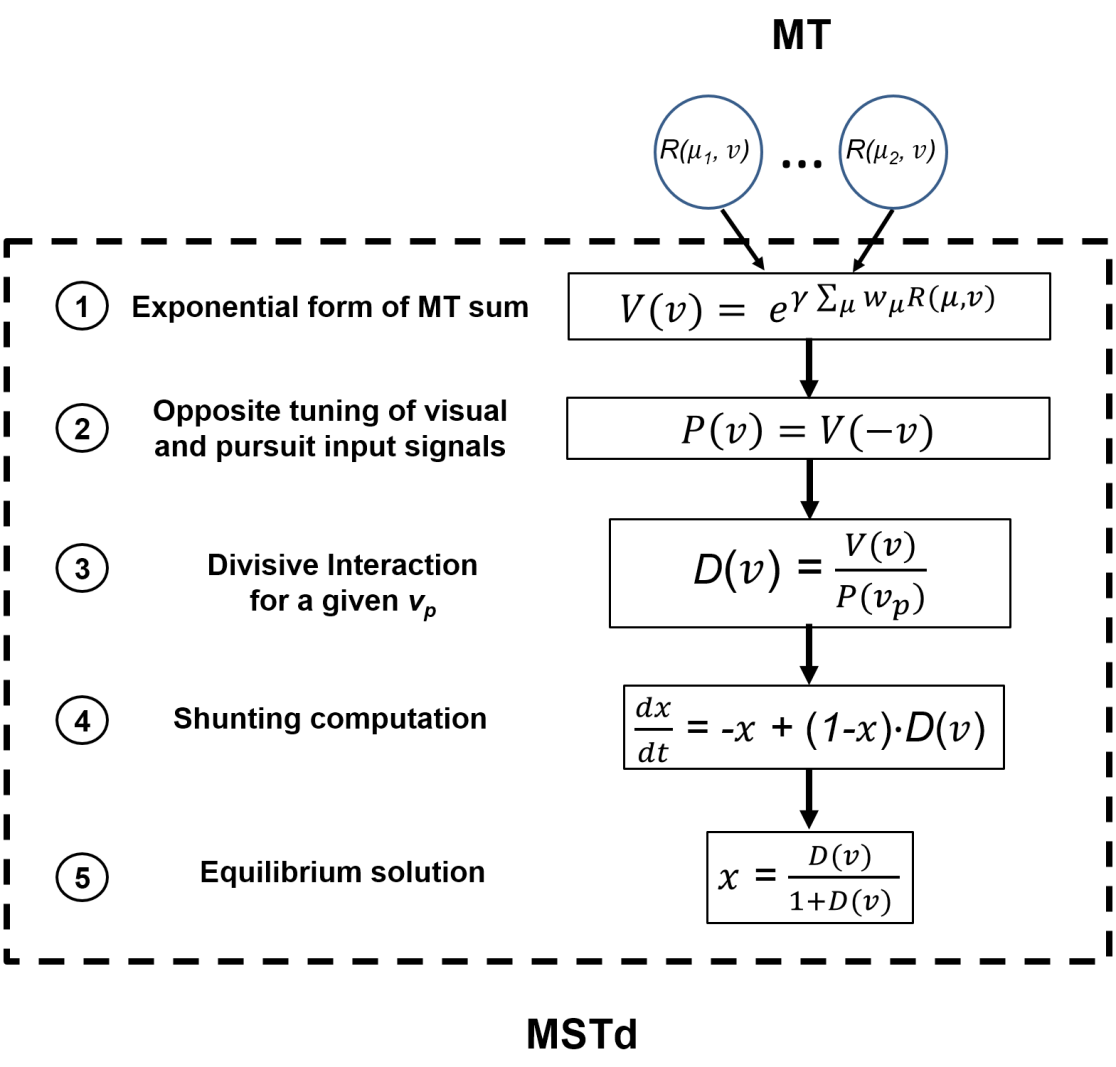
The log-Gaussian function of MT velocity tuning and the input to the model MSTd neuron. (a) Three examples of the velocity tuning curves of model MT neurons with the preferred velocity parameter *μ* set at 10, 20 and 30 degrees per second. (b) The summing weights follow a power function of preferred speed with a negative power. The response is assumed to be negative when the preferred direction of MT neurons is opposite to their projecting MSTd neuron. In each plot, the x-axis is the retinal velocity in degrees per second, and the y-axis is unit-less since the responses are normalized.

The output of an MT neuron, *R* is a log-Gaussian function of *q*, which is a function of the input retinal velocity, *v*, and the preferred velocity of the neuron in the preferred direction, *μ*, adjusted by a speed constant *s*_0_ to avoid infinite values for the logarithm function. We label a direction of visual motion as positive, if it is in the preferred direction of the model MSTd neuron, to which a given MT neuron projects. MT neurons are assumed to be silent (zero activity, or *R* is set to zero), when presented with their anti-preferred velocity. The standard deviation of the log-Gaussian function is defined by *σ* = 1.16, and the speed adjustment parameter *s*_0_ = 0.33, both of which are the medians of physiological recordings [7]. Fig 5a shows the speed tuning curves of log-Gaussian MT neurons with three different preferred velocities.

The weights for the log-Gaussian MT neurons are derived from a power distribution function *w*_*μ*_ = *μ* ^−0.1^ (see S1 File for details). The form of this reciprocal function is derived from the distribution of MT neurons with different preferred speeds as measured in [7], while the exact power (-0.1) is a free parameter and is determined by eye to qualitatively fit the sample MSTd neuron response in [6]as shown in Fig 3b. *w*_*μ*_ is negative, representing an inhibitory input, when the visual motion velocity is in the anti-preferred direction of the MSTd neuron that MT neurons project to. The sum of MT responses to visual motion along the preferred direction axis shows a sigmoidal shape as a function of velocity (Fig 5b).

The summation of MT responses is crucial for the sigmoidal shape of visual motion velocity tuning of the model MSTd neuron. The visual signal to the model MSTd neuron for different velocities, *V*, is the exponential form of the MT summation, scaled by the coefficient *γ* (Eq 4). The value of the scale coefficient (*γ* = 10) is chosen to fit the MSTd velocity tuning for different pursuit conditions.

$$V\left(v\right)\;=\;e^{\gamma R_{MT}}=e^{\gamma }\Sigma_{\mu }w_{\mu }R\left(\mu ,v\right)$$ (4)

The values of *γ* and the negative power in the weighting function of MT responses are the only two free parameters chosen to fit the model MSTd response to the physiological data.

Other than the visual input from MT neurons, the model MSTd neuron also receives the pursuit signal as part of the input. Although the origin of the pursuit signal is still not clear, the tuning of MSTd neurons to pursuit velocities can be very similar to their tuning to visual motion velocities [8]. Hence, in our model, the pursuit signal has the same velocity tuning shape as the visual signal, though the preferred pursuit direction can vary. To simplify the present implementation of our model while preserving its generality, we assume the preferred pursuit direction is either the same as or opposite to the preferred visual motion direction. For the pursuit compensation MSTd neuron, the input induced by pursuit (pursuit input), *P*, has an opposite velocity tuning compared to the visual input, meaning that the velocity tuning of pursuit input is a mirror image (hence opposite direction) of that of visual motion along the preferred direction axis for a given pursuit velocity *v*_*p*_ (Eq 5).

$$P\left(v_{p}\right)\;=\;V\left(-v_{p}\right)$$ (5)

The input to the model MSTd neuron, *D*, is the result of dividing the visual input by the pursuit input (Eq 6). The velocity in the visual input, *v*, and the velocity in the pursuit input, *v*_*p*_, are independent from each other. For a given pursuit velocity, *v*_*p*_, the result of the divisive interaction of *V* and *P* is a function of velocity of motion on retina, *v*.

$$D\left(v\right)\;=\;\frac{V\left(v\right)}{P\left(v_{p}\right)}$$ (6)

The opposite tuning of the pursuit input that serves as the denominator in the divisive interaction discounts the visual response when the pursuit is in the anti-preferred visual motion direction, while it enhances the visual response when the pursuit is in the preferred direction of visual motion. Divisive normalization is known to be useful in modeling visual processes [9]. However, the divisive interaction we use here involves signals from different modalities (the visual and pursuit inputs). This input is fed into a simplified shunting equation (Eq 7) to compute the output of the model MSTd neuron, *x*, by solving Eq 7 [10]. For simplicity, we solve Eq 7 in its equilibrium form (Eq 8), as the output of our model MSTd.

$$\frac{dx}{dt}=-x\;+\;\left(1-x\right)\cdot D\left(v\right)$$ (7)

$$\;x=\frac{D\left(v\right)}{1+D\left(v\right)}$$ (8)

The combination of the exponential input to the model MSTd neuron and shunting computation gives us the two desired properties of the MSTd velocity tuning simultaneously: (a) sigmoid-shape response curve, given the log-Gaussian inputs from MT, and (b) pursuit compensation. For (a), any supralinear input (a power function with a power greater than 1) can generate a bounded sigmoid-like response, given the equilibrium solution of the shunting equation (Eq 8). The supralinear excitation and power law of firing rates can explain many functional properties of the visual system [11,12]. However, we find that in our model the exponential function provides a much smoother shape of the output function than a power function in the given velocity range. The resulting output response function is very similar to the observed MSTd velocity tuning function. Furthermore, for the exponential function, the response amplitude is almost invariant for different pursuit conditions, while the response amplitude varies for the power function. For (b), pursuit compensation can be understood as a shifting property of the velocity tuning curve based on the pursuit velocity. The divisive interaction between the visual motion and pursuit inputs is a vector subtraction between these two modalities under exponential forms (consider $e^{s}/\;e^{s_{p}}=e^{s-s_{p}}$; however, the actual computation is slightly more complicated after the MT responses with different preferred velocities are weighted and summed). When the dynamics of the response is controlled by Eq 8, the visual velocity tuning of the output can shift its position depending on the pursuit velocity in the retinal coordinates without changing the response amplitude. The amount of the shift is the same as the amount of visual motion induced by pursuit in the retinal coordinate, and thus the visual velocity tuning of the model MSTd neuron is pursuit invariant. This pursuit invariant property is the pursuit compensation observed in [6]. Subtraction, instead of divisive and shunting interactions, does not work because it will change the overall amplitude instead of shifting the tuning curve (pursuit compensation).

## Results

### Pursuit compensation property of the model MSTd neuron

**Fig 6:**
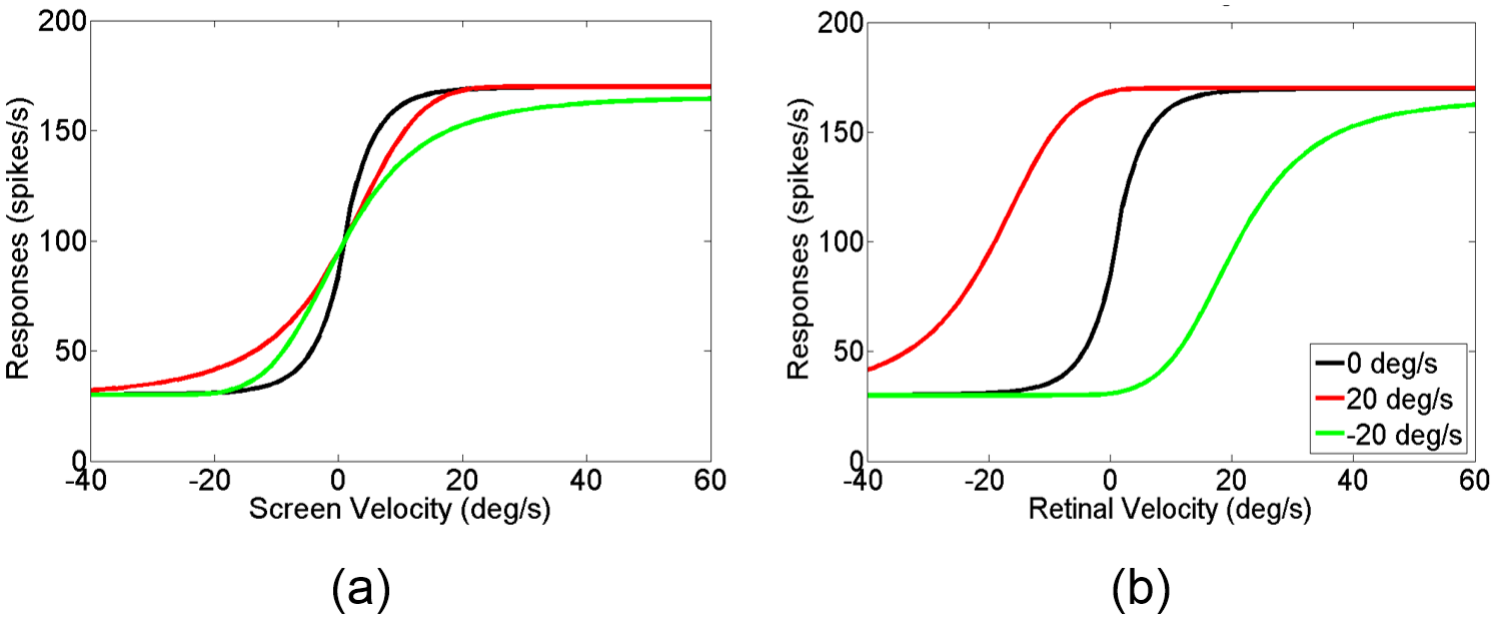
Model MSTd responses as a function of retinal visual velocity for different pursuit velocities compared to single function fit. The original MSTd velocity tuning data and the fitting function from Inaba et al. (2011) are shown in filled circle and solid line, respectively. The velocity tuning curve during the fixation, the pursuit to the preferred direction, and the pursuit to the anti-preferred direction conditions are shown in black, red, and green, respectively. For preferred pursuit direction (red lines), the relative intensity ratio of the visual motion signal to the pursuit signal is stronger than that for the anti-preferred pursuit direction (see text for details).

Fig 6 shows the model MSTd responses. Inaba et al. reported a curve-fit of MSTd responses with a mathematical function of velocity on the retina [6] (our Fig 3b). Our model uses the MT responses, not velocity, as the input to generate the simulated MSTd responses to different visual motion velocities under different pursuit velocities. Our model thus provides insight about the neural mechanisms of how MSTd neurons integrate visual and pursuit inputs as neural activities, such as divisive interaction of the neural signals from these two modalities.

The output of the model MSTd neuron shows the pursuit compensation property (Fig 6). If the pursuit is in the same direction as the preferred direction of the visual motion, the velocity tuning curve to visual motion shifts toward the left (red lines); if the pursuit is in the opposite direction to the preferred direction of the visual motion, the velocity tuning curve to visual motion shifts toward the right (green lines).

In the experimental data, there was a slight asymmetry between the velocity tuning of different pursuit directions for the same pursuit speed, e.g., the velocity tuning during pursuit in the preferred direction of visual motion (pursuit in the preferred direction) and the velocity tuning during pursuit in the opposite direction of the preferred visual motion direction (pursuit in the anti-preferred direction). Our model can simulate this asymmetry by adjusting the ratio of the pursuit signal weight to the visual motion signal weight. However, because it is not clear whether the pursuit signals for different pursuit direction affect the response of MSTd neurons differently, in the following discussion, the pursuit and visual motion signals contribute equally, and thus the ratio is 1.

## Discussion

In this study, we have developed a model to account for two main properties of typical pursuit MSTd neurons: the sigmoidal shape of the velocity tuning curve and the pursuit compensation response. The model has a feedforward structure and has only two fixed parameters to simulate MT velocity tuning and two free parameters for the exponential transformation of MT responses and the weighted summation of the resulted transformation. We next discuss the implications of the model. We also discuss how a pursuit compensation mechanism that operates only along the pursuit direction preferred by particular MTSd neurons can be extended to account for pursuit compensatory responses in all directions.

### Implications of our model

Our model links the velocity tuning of visual motion and pursuit with the pursuit compensatory property. Two implications can be derived from the computations of the model:

1) *Compensatory pursuit neurons in MSTd have similar velocity tuning curves of responses to pursuit and visual motion; it should be true as well that if a neuron in MSTd shows similar velocity tuning curves of pursuit and visual motion*, *this neuron should be a compensatory pursuit neuron*.

**Fig 7:**
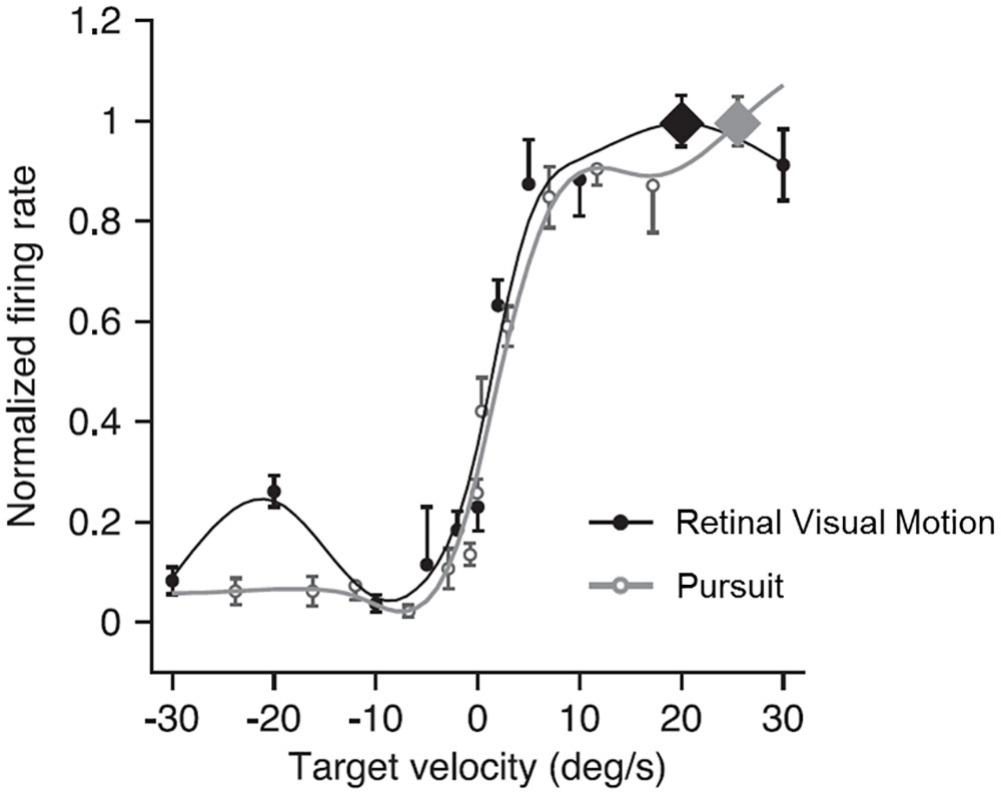
An example of a predicted compensatory pursuit neuron with similar velocity tuning of pursuit and visual motion. The black dots and line show the tuning and corresponding fit of visual motion, while the gray open dots and line show those of pursuit. Adapted from Churchland and Lisberger (2005).

This implication leads to a convenient way to find compensatory pursuit neurons experimentally. By measuring the velocity tuning of pursuit without visual motion and the velocity tuning of visual motion without pursuit, we can tell if the neuron is a compensatory pursuit neuron or not. To test the implication in an experiment, one should first measure the velocity tuning of a neuron’s responses to pursuit and visual motion, and then one should measure its velocity tuning of visual motion at different pursuit directions (the same or the opposite to the visual motion direction) similar to what was done in [6]. The implication is that if the neuron shows the same preferred direction of pursuit and visual motion, this neuron should also show the pursuit compensation property. In other words, the pursuit compensation property and the same preferred direction of pursuit and visual motion should be observed in one neuron simultaneously. As an example, we believe that the two neurons shown in [8] are compensatory pursuit neurons (Fig 7).

This claim may seem to be counter-intuitive at first glance, because the pursuit compensatory neuron in general prefers the same direction of pursuit and visual motion, while it also discounts the response to visual motion with the response to pursuit when both are present. However, the term “compensation” is used with the screen as the reference frame. The visual motion input to the neuron is the retinal visual motion, not the visual motion on screen. The screen motion can actually be derived from the vector summation of the retinal motion and the pursuit (for further discussion, refer to section “Component pursuit compensation on the single-cell level”). The MSTd neuron in our model implements this vector summation along the preferred direction axis in a neural response space by using divisive interaction between the visual motion and pursuit signals.

In reality, if either of the visual or pursuit input to the MSTd neuron is not in exponential form, or they do not interact in a divisive way perfectly, or they are imbalanced, we may observe a weak response in pursuit only, or an asymmetry, in tuning or in amplitude, of the pursuit compensation for different pursuit directions. A more thorough model with a population of neurons, each with a different balance of the visual and pursuit input, interacting with each other, may explain more complex patterns in data.

The second implication of the model is:

2) *There should exist neurons that show opposite preferred directions of pursuit and visual motion*. *Instead of compensating for pursuit*, *these neurons show a modulation of response induced by pursuit opposite to the compensatory pursuit neuron*, *meaning that the response to the preferred retinal visual motion is suppressed by the pursuit of the same direction*, *and is enhanced by the pursuit to the opposite direction*. *We name these neurons “non-compensatory pursuit neurons”*.

This implication of the model indicates that some pursuit neurons will show opposite velocity tuning of pursuit and visual motion. That is, these neurons have opposite preferred directions of pursuit and visual motion. Instead of compensating for pursuit induced motion on the retina, these neurons will show an enhanced response, when visual motion is in the preferred direction while the pursuit is in the opposite to the direction of visual motion (thus also the preferred pursuit direction). However, when the pursuit is in the same direction of the preferred visual motion, the neuron shows a much lower response than when there is no pursuit. So we call this type of neuron a non-compensatory pursuit neuron, as compared to the compensatory pursuit neuron. This type of neurons should be more sensitive to background motion induced by eye movements, and may interact with pursuit compensatory neurons for further eye movement planning.

**Fig 8:**
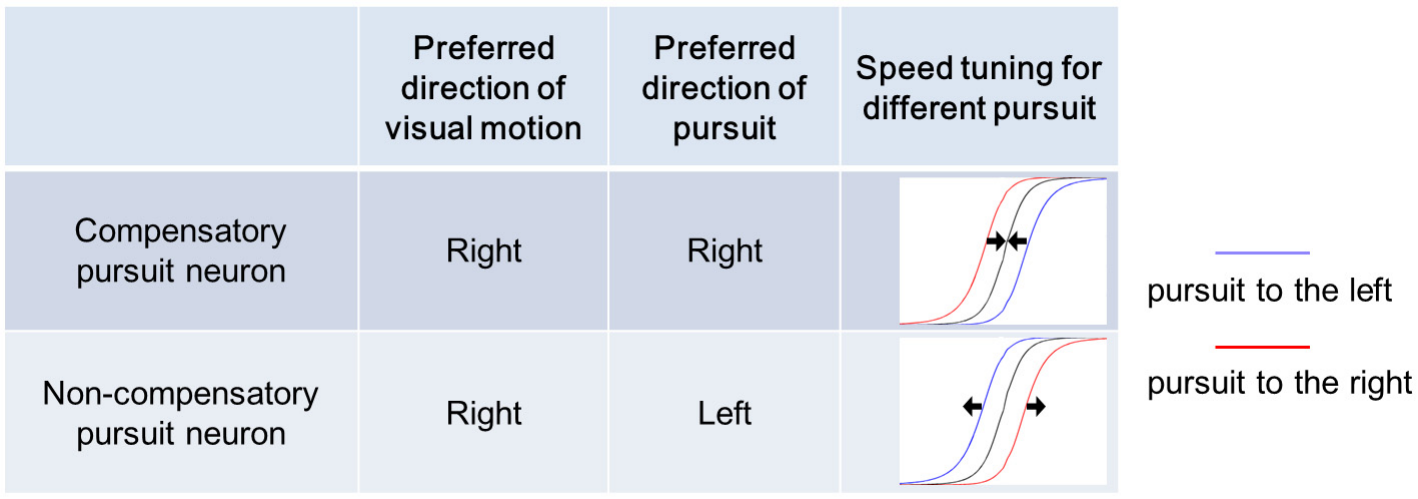
Different pursuit modulations of velocity tuning in compensatory and non-compensatory pursuit neurons. We assume both neurons prefer visual motion to the right. Correspondingly, the compensatory pursuit neuron should show a preferred direction of pursuit to the right, while the non-compensatory pursuit neuron should show a preferred direction of pursuit to the left. According to our analysis, if we know the neuron’s preferred direction of both pursuit and visual motion, we should be able to tell if the neuron is a compensatory pursuit neuron or a non-compensatory pursuit neuron. Diagrams of the retinal velocity tuning of visual motion during pursuit to the left and right directions are shown in Fig 8. Red lines represent pursuit to the right (preferred visual motion direction); blue lines represent pursuit to the left (null visual motion direction). The black arrows show the shift direction of these tuning curves when plotted against the screen velocity instead of the retinal velocity. The compensatory pursuit neuron compensates for the pursuit irrespective of the pursuit direction by converging the tuning curves in the screen frame, while the non-compensatory pursuit neuron spreads out the velocity tuning curves for different pursuit directions in the screen frame.

The differences between the pursuit compensation and non-compensatory pursuit neurons are shown in Fig 8, in which we assume the preferred direction of visual motion of the neurons is to the right. According to our analysis, if we know the neuron’s preferred direction of both pursuit and visual motion, we should be able to tell if the neuron is a compensatory pursuit neuron or a non-compensatory pursuit neuron. For example, if we know the neuron prefers right motion direction as well as right pursuit, we believe this neuron will be a compensatory pursuit neuron. The right column of the table also shows cartoons of the retinal velocity tuning of visual motion during pursuit to the left or right directions for both types of neurons. The black arrows show the shift direction of these tuning curves if we show these curves against the screen velocity instead of the retinal velocity. In the screen frame, the compensatory pursuit neuron shows overlapped velocity tuning curves of different pursuit velocities (shown by converging black arrows in the cartoon in Fig 8), while the non-compensatory pursuit neuron shows more spread-out velocity tuning curves for different pursuit velocities than in the retinal frame (shown by diverging black arrows in the cartoon in Fig 8).

The existence of non-compensatory pursuit neurons can explain the synergistic property of pursuit and visual motion responses in the neurons that have opposite direction preferences for pursuit and visual motion in [13]. These neurons may be involved in motion representation during self-movement, as independent components, similarly to the interaction of MT neurons with different properties during motion discrimination [14].

Komatsu and Wurtz observed that “the cells that prefer large-field stimuli in the lateral part of MST (MSTl) and MSTd usually show facilitation with movement of the background during pursuit. Most of these pursuit cells have opposite preferred directions for pursuit and visual motion.” [13] According to the model’s implications, this may seemingly lead one to consider that the majority of MSTd neurons are non-compensatory pursuit neurons, which is not consistent with the findings that the majority of the MSTd neurons are pursuit compensatory according to Inaba et al. [6,15]. This seemingly inconsistency is probably related to “a reversal of the preferred direction of motion as the size of the stimulus field increased” observed by Komatsu and Wurtz. Further, as they noted, “if we consider only the cells that show a reversal of preferred direction for large- compared to small-field stimuli, the preferred direction for the large field was always the opposite to that of pursuit, and the preferred direction for the small field was always the same” [13]. However, it is important to note that the stimulus size required to reverse the visual motion direction preference of these pursuit neurons increases with the visual motion speed. The reversal stimulus size can be as large as 73 degrees of visual angle with a visual motion speed of 28 degrees per second [13]. Thus, a significant portion of neurons that Komatsu and Wurtz observed might be actually compensatory pursuit neurons. With a different set of stimuli, Sakata et al. found that the pursuit neurons (visual tracking neurons in their study) with the same preferred direction of pursuit and visual motion and the pursuit neurons with the opposite preferred directions of pursuit and visual motion are about the same number [16]. However, the speed dependent spatial configuration of the preferred stimuli of MSTd neurons is out of the scope of the current study and will be investigated in the future. Further experiments considering stimulus size, pattern, speed interactions during pursuit are also necessary in order to better understand the interplay of these factors.

The concept of pursuit compensation refers to the compensation of the extra visual motion component on the retina induced by the pursuit. This compensation is a modulation of the visual motion response depending on the pursuit direction. For compensatory pursuit neurons, the modulation is excitatory when the pursuit is in the preferred visual motion direction, which we suggest is also its preferred pursuit direction; the modulation is suppressive when the pursuit is in the anti-preferred visual motion direction, which we suggest is also its anti-preferred pursuit direction. For non-compensatory pursuit neurons, the modulation is excitatory, when the pursuit is in the anti-preferred visual motion direction, which is actually its preferred pursuit direction; the modulation is suppressive, when the pursuit is in the preferred visual motion direction, which we suggest is actually its anti-preferred pursuit direction. So according to our analysis, we unify the pursuit response modulations for both groups of neurons: whenever the pursuit is in the neuron’s preferred pursuit direction, the visual response of this neuron is enhanced; whenever the pursuit is in the neuron’s anti-preferred pursuit direction, the response is suppressed.

### Component pursuit compensation on the single-cell level

**Fig 9:**
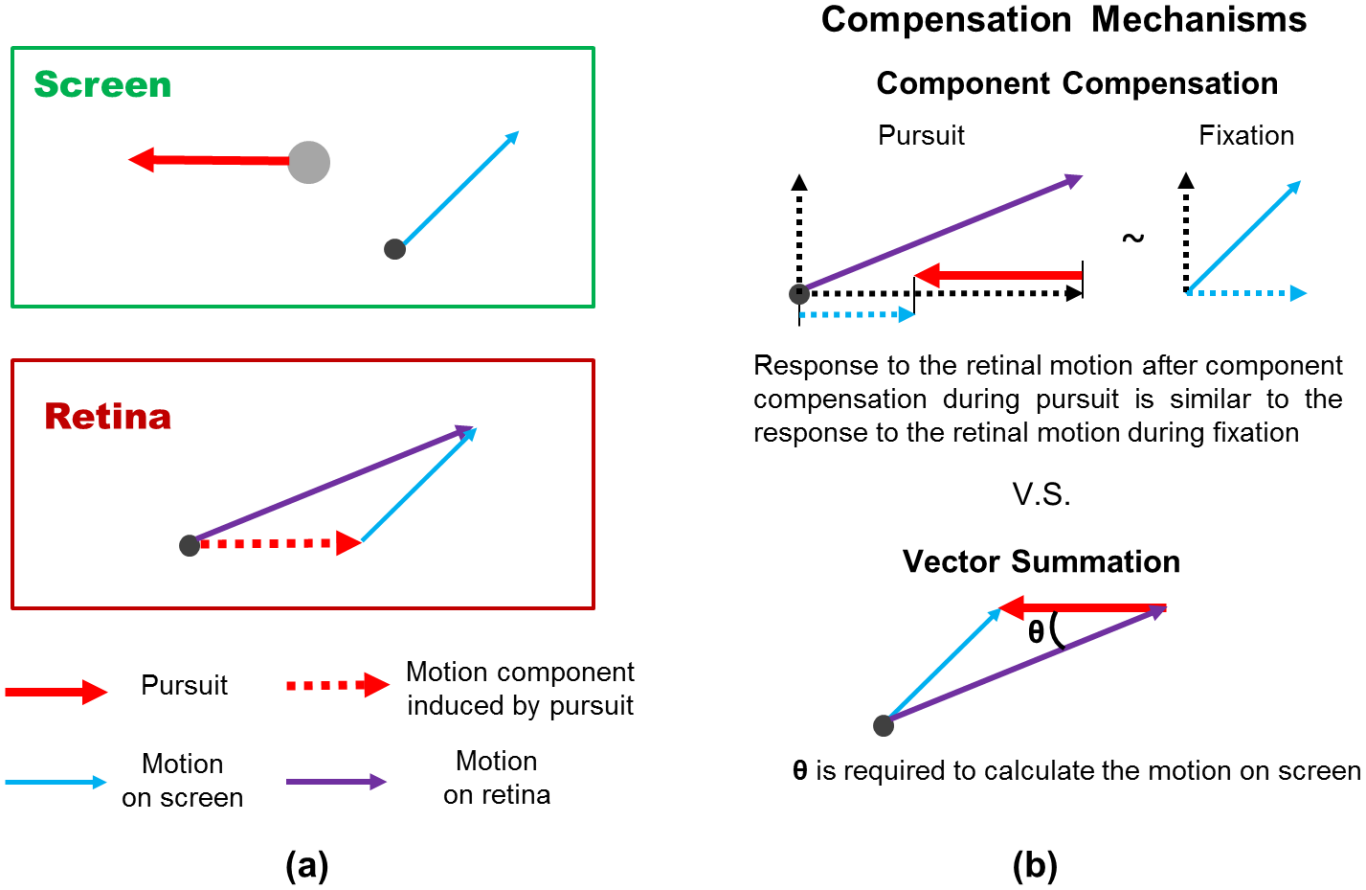
Component compensation versus vector summation as a single-cell level mechanism of pursuit compensation. **(a)** Pursuit (red arrow) induces a motion component (dashed red arrow) for the moving background (black dot). Thus, the motion on retina (purple arrow) is a vector sum of the motion on screen (blue arrow) and the motion component induced by the pursuit. A compensation mechanism can help the neuron respond to the same motion on screen similarly, whether there is pursuit or not. The goal is to code the motion on screen correctly, even when input from the retina is changed due to pursuit. **(b)** Two possible pursuit compensation mechanisms. Component compensation can be done by the mechanism suggested by our model. The MSTd neuron responds (horizontal dashed black arrow) to the motion input from MT (purple arrow). The response is adjusted by the pursuit input (red arrow). This adjustment can be achieved by the divisive interaction between the visual motion component along the preferred direction (horizontal dashed black arrow) and the pursuit (red arrow). Then, the MSTd neuron responds similarly to the same motion on screen, with or without pursuit (horizontal dashed blue arrows). In the component compensation approach, only the component of the actual motion on screen along the preferred direction is represented. However, this single MSTd neuron can show similar direction tuning under pursuit and fixation conditions (responses to dashed blue arrows). The vector summation mechanism can compensate for the pursuit and reveal both the speed and direction of the actual motion on screen. However, for the vector summation to work, the direction and speed of both the visual motion and pursuit vectors should be represented in neural responses, but the angle between these two vectors (**θ**) should also be represented in neural responses. Perfect representation of such information is difficult to implement in a single neuron. Thus, we suggest that component compensation can happen on a single-cell level in MSTd neurons first, so that the visual motion component along the neuron’s preferred direction can be represented. Then the population responses of these MSTd neurons with different preferred directions can be pooled together to represent precisely the actual motion on screen in the presence of pursuit eye movement.

The point of pursuit compensation is to reveal the actual motion of objects in the external world, even when the eye is pursuing a moving target. This is not a trivial task, because everything on the retina is affected by eye movements, and the image on retina, not the external motion, is what the brain receives. The motion on retina can be represented as the summation of two vectors: the actual screen motion vector and the motion vector induced by pursuit, which is in the opposite direction to the pursuit vector (Fig 9a). Thus, because MT neurons only receive retinal motion as input, they do not know the actual motion on screen. The output from these MT neurons, however, is the major visual input to the neurons that compute the pursuit compensation in MSTd.

An ideal pursuit compensation mechanism should compute the vector summation of the retinal motion vector and the pursuit vector, in order to compensate for the motion component induced by pursuit within the retinal motion (Fig 9b). However, this mechanism requires knowledge of the angle between the retinal motion vector and the pursuit vector, which is hard to implement on the single-cell level. Instead, we think the compensation can be readily computed by the neuron along its preferred direction, and thus only the component along the preferred direction is compensated for the pursuit. In our model, this component compensation is realized by projecting the visual input to the preferred direction of the model neuron and the divisive interaction between the convolved visual input and the pursuit input. This could be an easier way to achieve pursuit compensation on a single-cell level than the vector summation. For any given direction of pursuit, MSTd neurons with different preferred directions may show different response to a certain direction of visual motion. Each of them may only compensate for the pursuit component of visual motion along its preferred direction. However, the population of these neurons may be able to represent the actual motion on screen with a mechanism similarly to the vector summation (see [17]).

**Fig 10:**
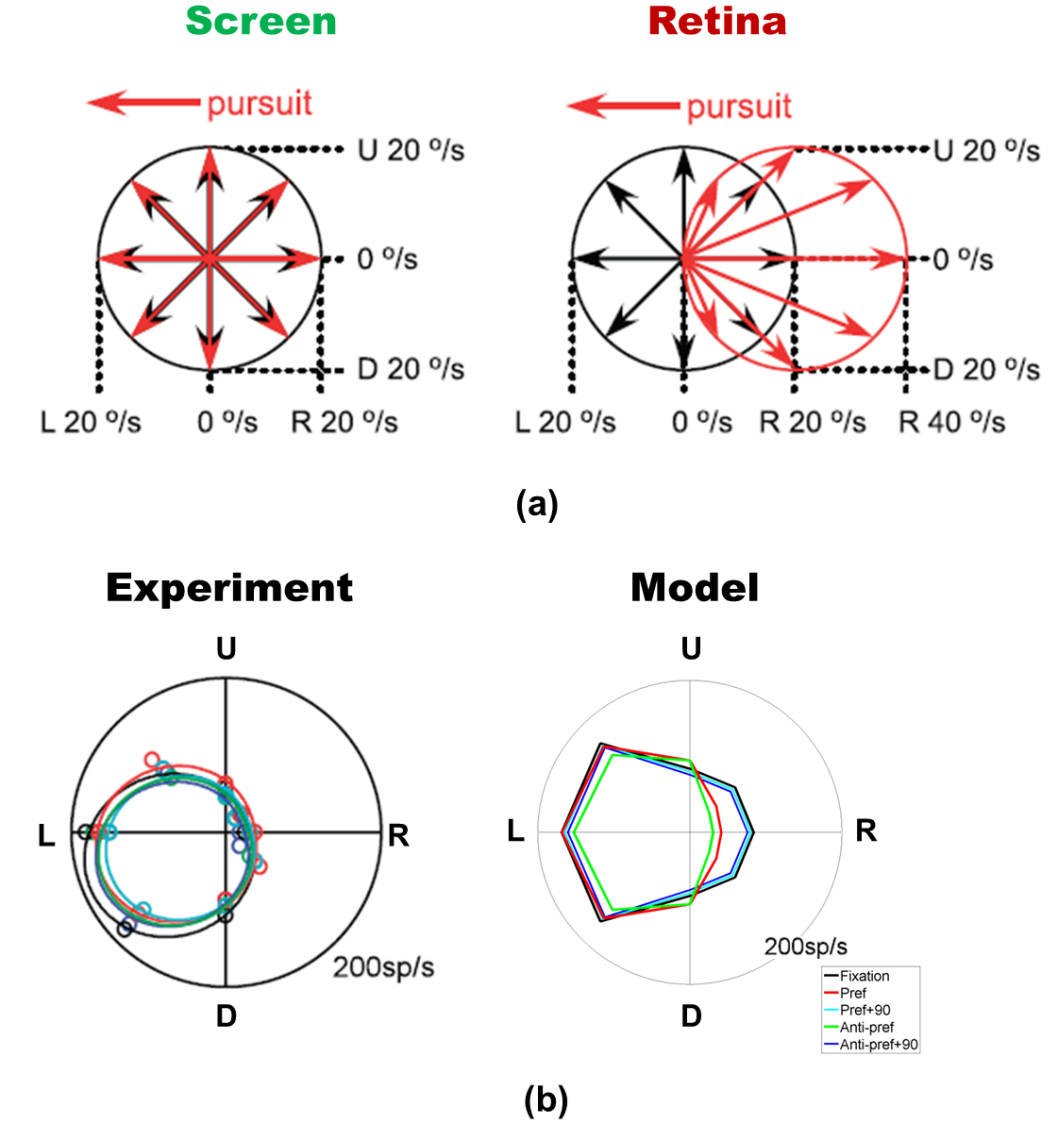
Direction-selective responses of the MSTd neuron shown in Fig 3 to the background motion on the screen during fixation (black arrows/lines) and pursuit in the preferred direction (red arrows/lines). **(a)** Motion on retina as a result of pursuit. Each arrow represents the motion of the background toward one of the eight directions. For the same background motion on screen for the fixation (black arrows) and pursuit conditions (red arrows), the motion on retina during pursuit is different from those during fixation. **(b)** The MSTd neuron shows similar direction tuning with or without pursuit. Our modeled neuron also shows similar direction tuning to the MSTd neuron during fixation (black line) and pursuit to preferred, preferred+90 degrees, anti-preferred, and anti-preferred+90 degrees direction (red, cyan, green and blue lines, respectively). (a) and the left plot in (b) are adapted from Inaba et al. (2011).

In Fig 10 we show that by using the idea of component compensation shown in Fig 9, we can replicate the direction tuning of experimentally measured MSTd neurons during fixation and pursuit to the preferred direction [6]. For the same visual motion pattern in different directions at 20 degrees per second on screen, a pursuit eye movement toward the left (the preferred pursuit direction) induced a visual motion pattern on retina, which was different from the pattern during fixation (Fig 10a). For this model MSTd neuron, all the visual motion signals were projected onto the preferred direction of pursuit (Fig 9b). Along the preferred direction axis of the model MSTd neuron, the model works just as described previously for each component from each direction. Consistent with the direction tuning of monkey MSTd neuron in [6], the resulting direction tuning for visual motion of our model MSTd neuron was almost invariant for different pursuit directions (Fig 10b).

### Limitations of our model

The current version of our model does not sample across spatial locations, and thus applies only to those MSTd neurons that are sensitive to planar motion that is uniform over space. Future studies can apply this model locally and integrate its responses over spatial locations, and thus address phenomena such as the perceived shifting of the focus of expansion in MSTd response to the expanding pattern and other optic flow patterns [18]. The model also does not take visual depth and perspective into account, which may generate different visual motion patterns that our brain could use to decode the effects of a pursuit eye movement without eye movement (motor) information. As noted by [19], retinal information for the cases of object motion and smooth pursuit of a static feature of the world by an eye rotation of a moving observer are not the same for a sufficiently wide view of a structured scene.

The current study models one class of MSTd neurons in each simulation to address the property of one representative neuron shown in [6]. Further studies can extend to a population of model neurons to accomplish the representation for complex visual motion patterns. However, data are limited with respect to the interaction between MSTd neurons.

## Conclusion

We have developed a model of pursuit compensatory MSTd neurons that employs divisive interactions of visual and oculomotor information. The model can explain the computational mechanisms of pursuit compensation. These mechanisms can be used in large-scale models, when visual and eye-movement inputs interact. We have discussed the implications of the model: (1) compensatory pursuit neurons in MSTd should have the same direction preference for pursuit and retinal visual motion; (2) there should be non-compensatory pursuit neurons that show opposite directional tuning for pursuit and retinal visual motion. These implications can be further experimentally tested. We have also discussed that on a single cell level, pursuit compensation responses to different visual motion and pursuit directions can be explained by a component compensation mechanism along a neuron’s preferred pursuit direction. Future experimental and theoretical studies based on this study may lead to a thorough understanding of visual motion and eye movement processing in our brain.

## Supporting Information

S1 File Distribution of preferred speeds in MT neurons. In this document, the different weighting functions to weight our model MT outputs are discussed.
(DOCX)

S1 Fig Normalized preferred speed distribution of MT neurons in linear (left) and logarithm (right) scales. A normalized power function (red lines) with a negative power, -1, can fit the data (black bars). The blue line shows *x* ^−0.1^ that is used as the weight of MT input to the MSTd neuron. Adapted from Nover et al. (2005).
(TIF)
