# Supplementary material for: Tuning Properties of MT and MSTd and Divisive Interactions for Eye-Movement Compensation
Source: PLoS One. 2015 Nov 17;10(11):e0142964. doi: 10.1371/journal.pone.0142964 (PMC4648577; doi:10.1371/journal.pone.0142964)
Supplement: S1 File — In this document, the different weighting functions to weight our model MT outputs are discussed. (DOCX) [file pone.0142964.s001.docx]

**Supplementary Materials**

**S1 File. Distribution of preferred speeds in MT neurons**

In this section, we will discuss the different weighting functions that our model used to weight MT outputs and that was reported by Nover et al. [1]. In our model the summing weights $w_{\mu}$ for the output of log-Gaussian MT neurons $R\left( \mu,v \right)$ are determined by $\mu^{-0.1}$, where $\mu$ is the preferred speed of the corresponding MT neuron. The sum of the weighted MT output determines the input to the model MSTd neuron. The weights thus represent the contribution of MT neurons with different preferred speed. The form of reciprocal function was derived from the distribution of MT neurons with different preferred speeds as measured in [1]. They suggest that the distribution of preferred speeds in MT should be logarithmically uniform (black bars, Figure S1, right). The uniform histogram in the logarithm scale can be mathematically fit by a power function with a negative power. In Figure S1, we show this fitting function (red lines) both in the linear and logarithm scale over the same physiological data [1]. In our model, the weights are chosen according to a similar power function with a negative power. Instead of -1 in power, we use -0.1 to fit the sample MSTd response as shown in Inaba et al. [2]. Note that it is still unknown whether the distribution of the preferred speed in MT neurons represents the exact contribution of their input to MSTd. It is possible that an MT neuron preferring a higher speed affects the target MSTd neuron more than an MT neuron preferring a lower speed. Furthermore, it is not clear that the proportion of MT neurons preferred speeds higher than 35 degrees per second is well predicted by the power function with a power of -1.


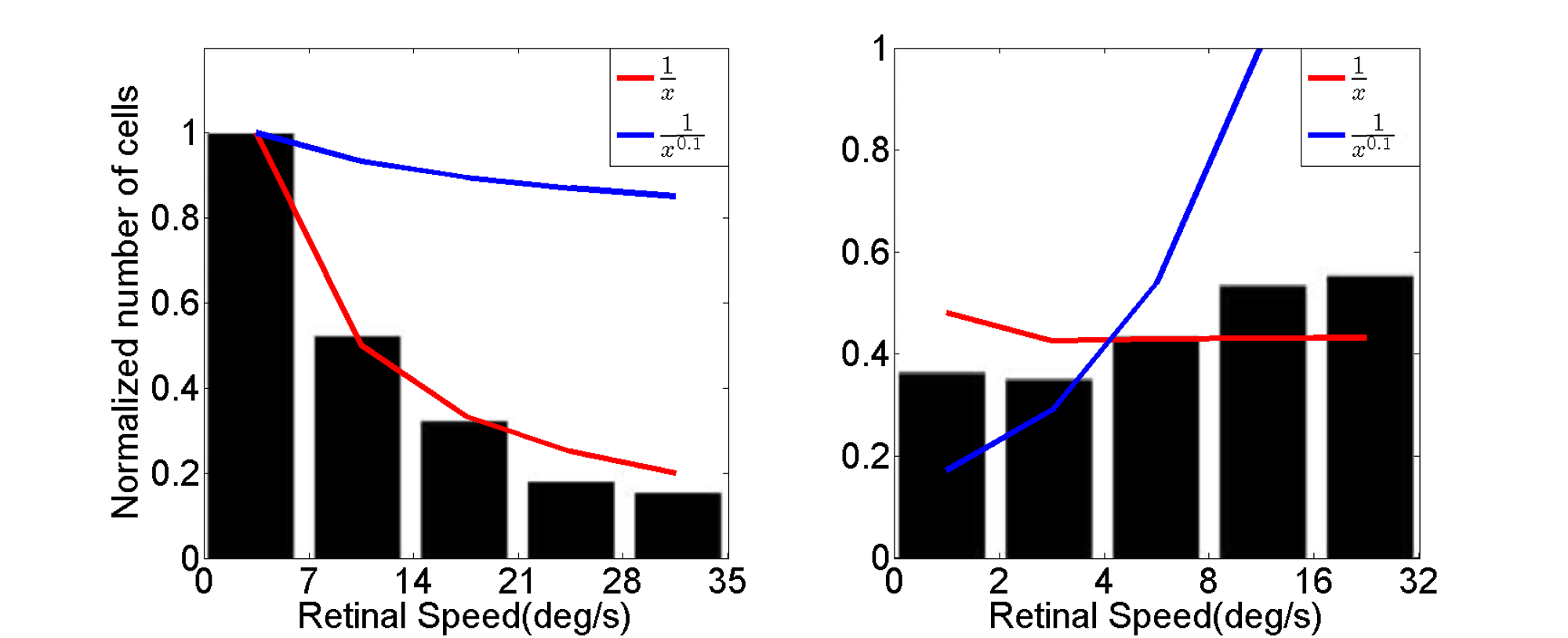


**Figure S1. Normalized preferred speed distribution of MT neurons in linear (left) and logarithm (right) scales.** A normalized power function (red lines) with a negative power, -1, can fit the data (black bars). The blue line shows $x^{-0.1}$ that is used as the weight of MT input to the MSTd neuron. Adapted from Nover et al. (2005).
