# Supplementary figures and images for: Tuning Properties of MT and MSTd and Divisive Interactions for Eye-Movement Compensation
Source: PLoS One. 2015 Nov 17;10(11):e0142964. doi: 10.1371/journal.pone.0142964 (PMC4648577; doi:10.1371/journal.pone.0142964)

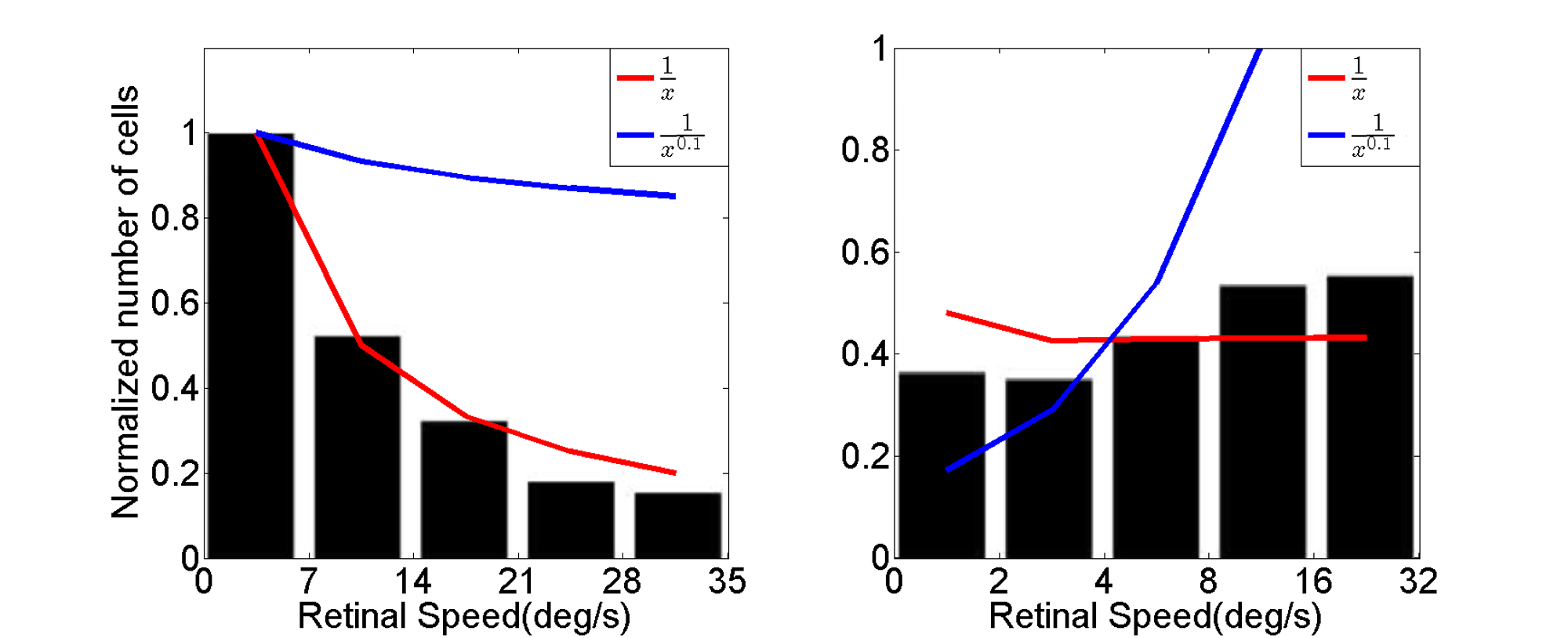

Supplement: S1 Fig — A normalized power function (red lines) with a negative power, -1, can fit the data (black bars). The blue line shows x −0.1 that is used as the weight of MT input to the MSTd neuron. Adapted from Nover et al. (2005). (TIF) [file pone.0142964.s002.tif]
